# Supplementary material for: Infection via mosquito bite alters Zika virus tissue tropism and replication kinetics in rhesus macaques
Source: Nat Commun. 2017 Dec 13;8:2096. doi: 10.1038/s41467-017-02222-8 (PMC5727388; doi:10.1038/s41467-017-02222-8)
Supplement: Supplementary file 1 — Supplementary Information [file 41467_2017_2222_MOESM1_ESM.pdf]

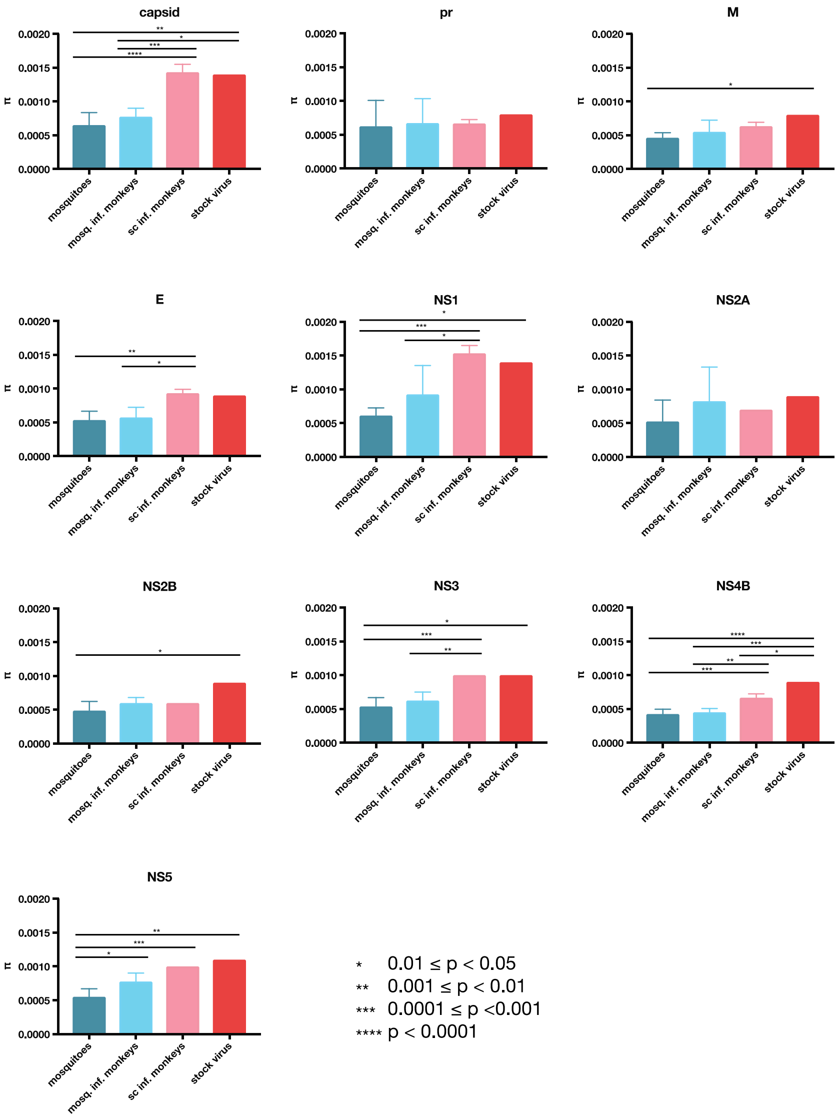

Supplementary Figure 1. Genewise nucleotide diversity (π) in viruses infecting monkeys and mosquitoes. We calculated π for each viral gene (except for protein 2K and NS4A, which did not have sufficient coverage in some samples) in viruses infecting each experimental group. Bars indicate mean and error bars indicate standard deviation, which are measured for each experimental group. Asterisks indicate statistical significance (one-way ANOVA with Tukey's correction for multiple comparisons).

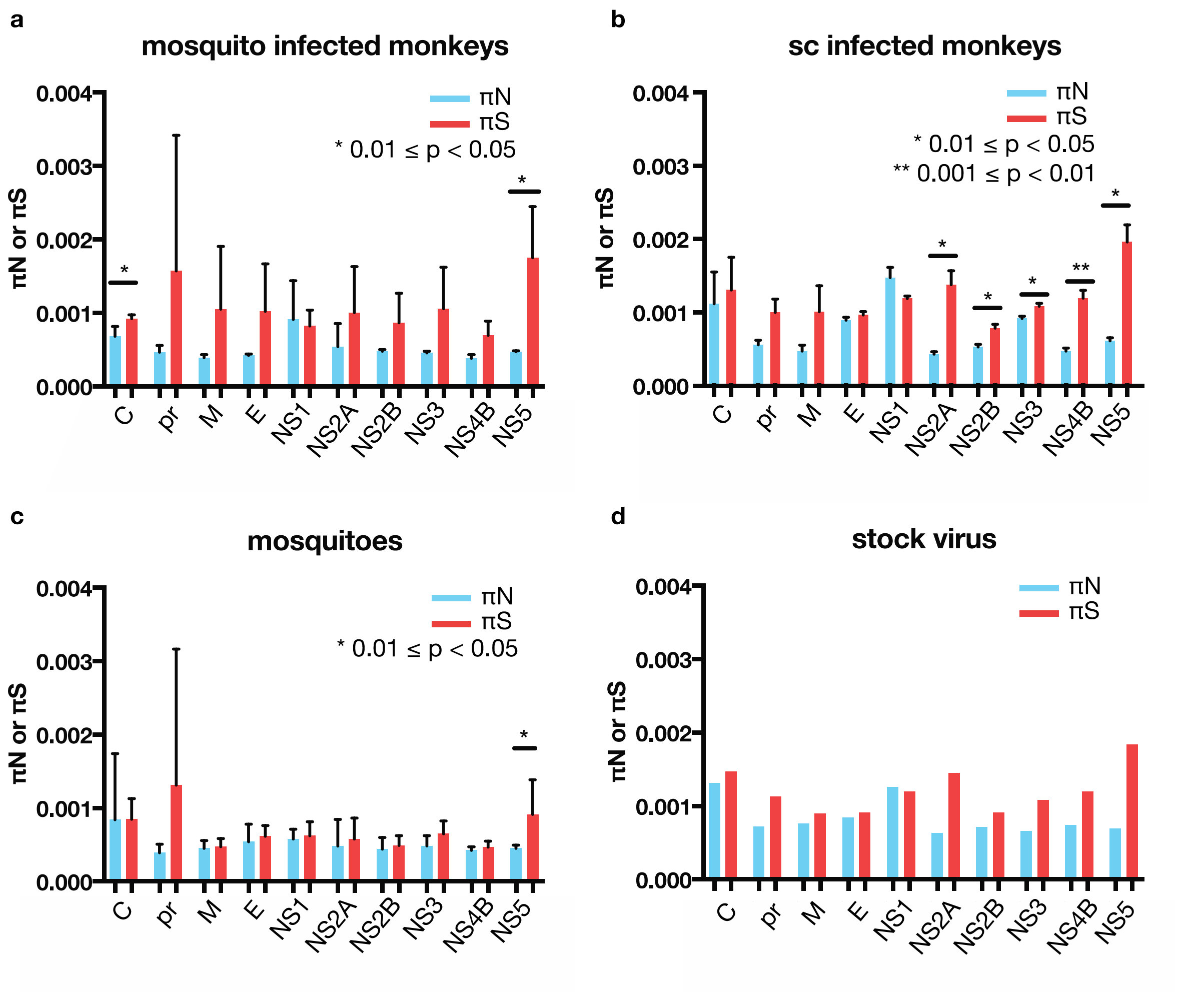

Supplementary Figure 2. Genewise synonymous and nonsynonymous nucleotide diversity (πS and πN) in viruses infecting monkeys and mosquitoes. We calculated πS and πN for each viral gene (except for protein 2K and NS4A, which did not have sufficient coverage in some samples) in viruses infecting each experimental group. Bars indicate mean and error bars indicate standard deviation, which are measured for each experimental group. For each gene, we determined whether πS was significantly different from πN as a test of the strength and direction of natural selection. Asterisks indicate statistical significance (Student's paired t-test).

Supplementary Table 1. ZIKV NS1-specific antibody measured by enzyme-linked immunosorbent assay (ELISA).

		Semi-Quantitative IgM Titer (Average OD Ratio)					
Infection Method	Animal ID	0DPI	7DPI	8DPI	9DPI	10DPI	14DPI
Subcutaneous	634675	nd	nd	nd	nd	nd	1.238
	566628	nd	nd	nd	nd	nd	3.074
	311413	nd	nd	nd	nd	nd	2.473
Mosquito Bite	268283	nd	nd	nd	nd	nd	4.270
	328696	nd	nd	nd	nd	nd	2.025
	458001	nd	nd	nd	nd	1.321	3.074
	349332	nd	nd	nd	nd	nd	2.424

Supplementary Table 2. Nucleotide variants in ZIKV challenge stock used to infect rhesus macaques.

Site relative to KU501215	Change	Amino Acid Change	Variant Frequency
1964	G->T	Val -> Leu	91.5%
2780	T->G	Trp -> Gly	5.8%
3147	T->C	Met -> Thr	10.2%
5679	C->T	Ser -> Phe	66.4%
7915	C->T	Synonymous	9.1%

*Only variants found in >5% of sequences in duplicate sequencing reactions are shown

Supplementary Table 3. Total viral template numbers added to cDNA synthesis reaction in sequencing pipeline.

Mosquito Saliva/Primate Plasma Sample	Day of Peak Viremia	Templates / cDNA Reaction in Replicate A	Templates / cDNA Reaction in Replicate B
Saliva-6	-------	426.8	345.3
Saliva-8	-------	21868.0	21868.0
Saliva-9	-------	10252.0	10252.0
Saliva-19	-------	1496.0	1496.0
Saliva-20	-------	368.7	368.7
Saliva-25	-------	2477.2	2477.2
Saliva-26	-------	43120.0	43120.0
Saliva-32	-------	293.9	293.9
328696	Day 5	43273.2	43273.2
458001	Day 5	52852.9	52852.9
349332	Day 5	3501.5	3501.5
268283	Day 6	64744.7	64744.7
566628	Day 3	5597.6	5597.6
311413	Day 3	23123.1	23123.1
